# Latest Arrivals in London Hospitals

**Published:** 1914-10-17

**Authors:** 


### HOSPITALS AND THE WAR.

(From our Special Correspondents.)

### Latest Arrivals in London Hospitals.

### THE WOUNDED AT ST. GEORGE'S HOSPITAL.

On Friday, October 9, in the early afternoon, the first batch of sick and wounded from the Front were admitted into this hospital.

Two of the wards of the hospital have been set aside for their use and form a military unit, cut off from the rest of the wards and under special regulations. This military unit of fifty beds may be considered as a branch of the Queen Alexandra Military Hospital, and the men are under military discipline, being in the charge of members of the staff who hold commissions in the Territorial branch of the Royal Army Medical Corps.

Fifty more beds are available when required at the convalescent hospital at Wimbledon. These will be called upon as required, so that the total number of 100 will ultimately be under treatment. The majority of the wounds are not very severe, and many of the men will soon be able to return to active duty. It is, therefore, hoped that the authorities will keep up the number so as to have all the beds allotted continuously filled.

### CROYDON.

The two wards offered to the War Office for the treatment of sick and wounded from the war have not yet been made use of, but cases from the Royal Naval School at the Crystal Palace are, in pursuance of an arrangement come to with the Admiralty, being received at the Croydon General Hospital. Many cases of minor accident and sickness among the Territorial recruits in Croydon and its vicinity are also being treated daily in various parts of the hospital—the main portion of them in the casualty surgeries and some in the wards—to their great comfort and satisfaction.

